# miR-27b-mediated suppression of aquaporin-11 expression in hepatocytes reduces HCV genomic RNA levels but not viral titers

**DOI:** 10.1186/s12985-019-1160-6

**Published:** 2019-05-02

**Authors:** Fuminori Sakurai, Rina Hashimoto, Chieko Inoue, Keisaku Wakabayashi, Tomohito Tsukamoto, Tsutomu Imaizumi, Taracena Gandara Marcos Andres, Eiko Sakai, Kanae Itsuki, Naoya Sakamoto, Takaji Wakita, Hiroyuki Mizuguchi

**Affiliations:** 1 Laboratory of Biochemistry and Molecular Biology, Graduate School of Pharmaceutical Sciences, Osaka University, 1-6 Yamadaoka, Suita, Osaka 565-0871 Japan; 2 Department of Gastroenterology and Hepatology, Graduate School of Medicine, Hokkaido University, North 15, West 7, Kita-ku, Sapporo, Hokkaido 060-8638 Japan; 3 Department of Virology 2, National Institute of Infectious Diseases, Toyama 1-23-1, Tokyo, Shinjuku-ku 162-8640 Japan; 4 Laboratory of Hepatocyte Regulation, National Institute of Biomedical Innovation, Health and Nutrition, 7-6-8, Saito-Asagi, Ibaraki City, Osaka 567-0085 Japan; 5 Global Center for Medical Engineering and Informatics, Osaka University, 2-2 Yamadaoka, Suita, Osaka 565-0871 Japan

**Keywords:** microRNA, HCV, miR-27b, Aquaporin-11

## Abstract

**Background:**

MicroRNAs (miRNAs) have gained much attention as cellular factors regulating hepatitis C virus (HCV) infection. miR-27b has been shown to regulate HCV infection in the hepatocytes via various mechanisms that have not been fully elucidated. In this study, therefore, we examined the mechanisms of miR-27b-mediated regulation of HCV infection.

**Methods:**

In silico screening analysis, transfection with miR-27b mimic, and a cell-based reporter assay were performed to identify miR-27b target genes. Cell cultured-derived HCV (HCVcc) was added to Huh7.5.1 cells knocked down for aquaporin (AQP) 11 (AQP11) and overexpressing AQP11. HCV replication levels were evaluated by real-time RT-PCR analysis of HCVcc genome.

**Results:**

Infection of Huh7.5.1 cells with HCVcc resulted in significant elevation in miR-27b expression levels. In silico analysis revealed that AQP11, which is an AQP family member and is mainly localized in the endoplasmic reticulum (ER), was a candidate for a target gene of miR-27b. Transfection of a miR-27b mimic significantly reduced AQP11 expression, but a cell-based reporter assay demonstrated that miR-27b did not suppress the expression of a reporter gene containing the 3′-untranslated region of the AQP11 gene, suggesting that miR-27b indirectly suppressed AQP11 expression. AQP11 expression levels were significantly reduced by infection with HCVcc in Huh7.5.1 cells. Knockdown and over-expression of AQP11 significantly reduced and increased HCVcc genome levels in the cells following infection, respectively, however, AQP11 knockdown did not show significant effects on the HCVcc titers in the culture supernatants.

**Conclusions:**

These results indicated that HCV infection induced a miR-27b-mediated reduction in AQP11 expression, leading to a modest reduction in HCV genome levels in the cells, not HCV titers in the culture supernatants.

## Background

Hepatitis C virus (HCV) is a single-stranded positive RNA virus that causes chronic liver diseases, including cirrhosis, and hepatocellular carcinoma. It is estimated that more than 70 million people worldwide are chronically infected with HCV. Currently, no vaccine for HCV is available. The combination therapy of pegylated interferon (IFN) plus ribavirin eliminates HCV from the liver in only a subset of HCV patients. Recently, combined therapies using direct-acting antivirus (DAA) agents, including Daclatasvir, Simeprevir, and Sofosubvir, have been shown to be effective [1, 2]; however, HCV variants resistant to DAA-based therapy have been reported [3, 4]. It is crucial to further clarify the infection process and pathogenesis of HCV in order to identify novel drug targets for effective therapy and to develop novel methods of hepatitis C treatment and prevention.

Recently, microRNAs (miRNAs) have attracted much attention as cellular factors controlling HCV infection [5–7]. The most notable miRNA in this capacity is miR-122a, which is a hepatocyte-specific miRNA [8]. miR-122a binds to the sites in the 5′-untranslated region (UTR) of the HCV genome and positively regulates the viral life cycle by enhancing viral RNA stability, translation, and replication, although the precise mechanism remains to be understood. In addition to miR-122a, several other miRNAs have been reported to play a role in HCV infection and pathogenesis, including miR-27a/b, miR-125b, miR-130a, miR-146a, and miR-181a [9–13]. These miRNAs positively or negatively regulate HCV infection and pathogenesis by suppressing the expression of host target genes, rather than by binding to the HCV genome. Therefore, the identification of target genes of these miRNAs would directly lead to an understanding of the process of HCV infection process and pathogenesis and the identification of novel target genes of anti-HCV drugs.

In this study, we focused on miR-27b, which is abundantly expressed in the liver [14], as a regulatory miRNA in the HCV life cycle. Previous studies demonstrated that miR-27b expression was elevated by HCV infection, and that miR-27b regulates lipid homeostasis by suppressing the expression of several genes, including peroxisome proliferator-activated receptor (PPAR)-α and angiopoietin-like protein 3 (ANGPTL3) [9, 15, 16]. However, it remained to be fully elucidated how miR-27b regulated the HCV life cycle and pathogenesis. This study demonstrated that miR-27b indirectly suppressed the expression of aquaporin (AQP)-11 (AQP11). AQP11 is an intracellular aquaporin family member involved in water and glycerol channel transport, although its precise functions remain unclear. Down-regulation of AQP11 resulted in a reduction in HCV genome copy numbers in Huh7.5.1 cells, while over-expression of AQP11 led to an increase in HCV genome copy numbers. These data suggested that AQP11 is a novel cellular factor positively regulating the HCV life cycle.

## Methods

### Cells

HEK293 cells (a human embryonic kidney cell line), Huh7.5.1 cells, which are a subclone of Huh7.5 cells and more permissive to HCV infection than Huh7 cells, and Huh7.5.1 1bFeo cells, which is a genotype 1b HCV replicon cell line [17], were cultured with Dulbecco’s Modified Eagle’s medium (DMEM) (Wako, Osaka, Japan) supplemented with 10% fetal bovine serum (FBS) and 1% penicillin/streptomycin at 37 °C in a 5% CO_2_ atmosphere.

### Infection with HCVcc

Cell culture-grown HCV (HCVcc, genotype 2a JFH-1 strain) was propagated in Huh7.5.1 cells as previously described [18]. Huh7.5.1 cells were seeded on a 12-well plate at a density of 5 × 10^4^ cells/well. On the following day, cells were infected with HCVcc at the indicated multiplicities of infection (MOIs). The medium containing HCVcc was replaced with fresh medium 6 h after infection. Total RNA and protein were recovered at the indicated time points, followed by real-time RT-PCR analysis and western blot analysis, respectively, as described below.

### Transfection with miRNA mimic, siRNA, antagomir, and plasmid DNA

Cells were transfected with miR-27b mimic, control mimic (GE Healthcare, Lafayette, CO), antagomir against miR-27b (antagomiR-27b) and control antagomir (Life Technologies, Carlsbad, CA) using Lipofectamine RNAiMAX (Life Technologies, Carlsbad, CA) on the day after cell seeding, followed by real-time RT-PCR analysis and western blot analysis as described below. In HCVcc infection experiments, cells were transfected with miR-27b mimic and control mimic as described above. Following a 24-h incubation, cells were infected with HCVcc at an MOI of 1. HCVcc genome copy numbers in the cells were determined by real-time RT-PCR analysis 72 h after infection. For knockdown of AQP11, cells were transfected with an siRNA against AQP11 (siAQP11) (Dharmacon SMARTpool;, GE Healthcare) or a control siRNA (siControl) (Dharmacon siGENOME Non-targeting siRNA Pool; GE Healthcare) at the indicated concentrations using Lipofectamine RNAiMAX reagent. For over-expression of AQP11, AQP11-expressing plasmid (pAQP11, see below) or a control plasmid (pcDNA3.1/Hygro(+); Life Technologies) was transfected in the cells at a concentration of 6 μg/ml using Lipofectamine 2000 reagent (Life Technologies). HCVcc was added to the cells 2 days after transfection, followed by real-time RT-PCR analysis.

### Real-time RT-PCR analysis

Real-time RT-PCR analysis of intracellular HCV RNA genome levels was performed as previously described [18]. Expression levels of miR-27b were determined as follows. In brief, total RNA was extracted from the cells using ISOGEN (Wako Pure Chemical). Reverse transcription was performed using a TaqMan MicroRNA Reverse Transcription Kit specific for hsa-miR-27b (Applied Biosystems, Foster City, CA). miR-27b expression levels were determined by using a Taqman Real-time RT-PCR system and normalized by the U6 snRNA expression levels.

### Plasmid DNA

The human AQP11-expressing plasmid DNA (pAQP11) was constructed as follows. The DNA fragment encoding the AQP11 protein was amplified by PCR using cDNA prepared from Huh7.5.1 cells. The fragment was then cloned into the multicloning site of pcDNA3.1/Hygro(+), resulting in pAQP11. The reporter plasmid psiCHECK-2-AQP11–3’UTR, which has the sequence of the wild-type 3′-UTR of the AQP11 gene downstream of the renilla luciferase gene, was constructed as follows. The approximately 270-bp fragment encoding the 3′-UTR of the AQP11 gene was amplified by PCR using cDNA prepared from Huh7.5.1 cells. The 3′-UTR sequence of the AQP11 gene contains the miR-27b-target sequence which is predicted by TargetScan [19]. The PCR fragments were ligated with a *Pme*I-digested fragment of psiCHECK-2 (Promega, Madison, WI), resulting in psiCHECK-2-AQP11–3’UTR. The positive control plasmid psiCHECK-2-control was constructed by insertion of oligonucleotides encoding the 2 copies of sequences perfectly complementary to miR-27b downstream of the renilla luciferase gene. The sequences of the primers and oligonucleotides used in this study are available on request.

### Reporter assay

HEK293 cells were seeded on a 12-well plate at a density of 5 × 10^4^ cells/well. On the following day, cells were co-transfected with the reporter plasmids and miR-27b mimic at the final concentration of 1 μg/ml and 25 nM, respectively, using Lipofectamine2000 reagent. After a 48-h incubation, renilla and firefly luciferase activities in the cells were determined using a Dual Luciferase Reporter Assay System (Promega, Madison, WI).

### Western blotting

Total cell lysates were resolved on 10% polyacrylamide gels (20 μg/lane). Samples were run at 20 mA for 1 h in 25 mM Tris buffer/0.2 M glycine/0.1% SDS buffer. Samples were then transferred to Immobilon-P (PVDF membrane; Millipore, Bedford, MA) for 2 h at 100 V in 25 mM Tris buffer/20% methanol. Blots were blocked with 5% skim milk for 1 h, incubated with polyclonal rabbit anti-AQP11 antibody (1:1000; Alpha Diagnostic International Inc., San Antonio, TX) for overnight, and then probed with the secondary antibody, mouse anti-rabbit IgG-horseradish peroxidase (1:5000; Cell Signaling Technology, Beverly, MA) for 30 min. Western blot analyses of HCV NS5A and GAPDH protein levels were carried out using mouse anti-NS5A antibody (9E10; kindly provided by Dr. Charles Rice, Rockefeller University, NY) and rabbit anti-GAPDH antibody (R&D Systems Inc., Minneapolis, MN), respectively. The bands were visualized by using an ECL plus detection kit (GE Healthcare) and analyzed using an LAS-3000 imager (Fujifilm, Tokyo, Japan).

### Cell viability

Huh7.5.1 cells were seeded on a 96-well plate at a density of 1 × 10^4^ cells/well. On the following day, cells were transfected with siAQP11 and control siRNA at the indicated concentrations. Cell viabilities were determined by an Alamar blue assay 3 days after transfection.

### Statistical analysis

Statistical significance was determined using Student’s *t*-test. Data are presented as the means ± S.D.

## Results

### Infection with HCVcc induced miR-27b expression in Huh7.5.1 cells

**Fig. 1:**
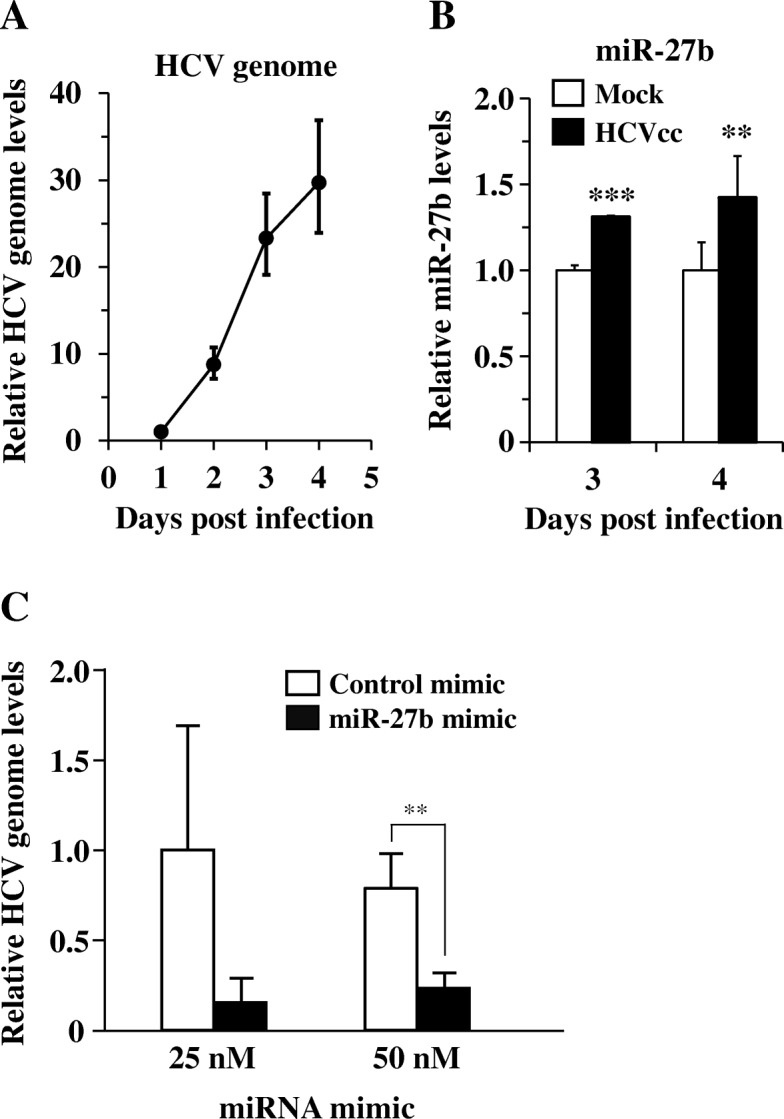
Increase in miR-27b expression following infection with HCVcc in Huh7.5.1 cells. **a** HCVcc genome copy numbers in Huh7.5.1 cells following infection. HCVcc genome copy numbers were normalized to GAPDH. **b** miR-27b expression levels in Huh7.5.1 cells following infection. Huh7.5.1 cells were infected with HCVcc at an MOI of 1. Total RNA was recovered from the cells at the indicated time points, followed by real-time RT-PCR analysis. miR-27b expression levels were normalized to U6. Expression levels relative to mock-infected cells are shown. **c** HCVcc genome copy numbers in Huh7.5.1 cells following transfection with miR-27b mimic. HCVcc genome copy numbers were determined by real-time PCR analysis 72 h after infection. HCVcc genome copy numbers were normalized to GAPDH. The relative HCVcc genome copy numbers in the cells transfected with 25 nM of control mimic were normalized to 1. The data are expressed as the means ± S.D. (*N* = 3). **p < 0.01, ***p < 0.001

First, in order to examine whether HCVcc infection alters the miR-27b expression profile, miR-27b expression levels in Huh7.5.1 cells were determined following infection by real-time RT-PCR analysis. The results showed that HCVcc was efficiently replicated in Huh7.5.1 cells (Fig. 1a). Infection with HCVcc resulted in approximately 1.3-fold and 1.4-fold increases in miR-27b expression levels at 3 and 4 days after infection, respectively (Fig. 1b). HCVcc genome levels were significantly reduced following miR-27b mimic transfection at 50 nM (Fig. 1c), indicating that miR-27b repressed HCV infection. These results indicated that infection with HCV induces miR-27b expression in the hepatocytes.

### miR-27b indirectly suppressed AQP11 expression

**Fig. 2:**
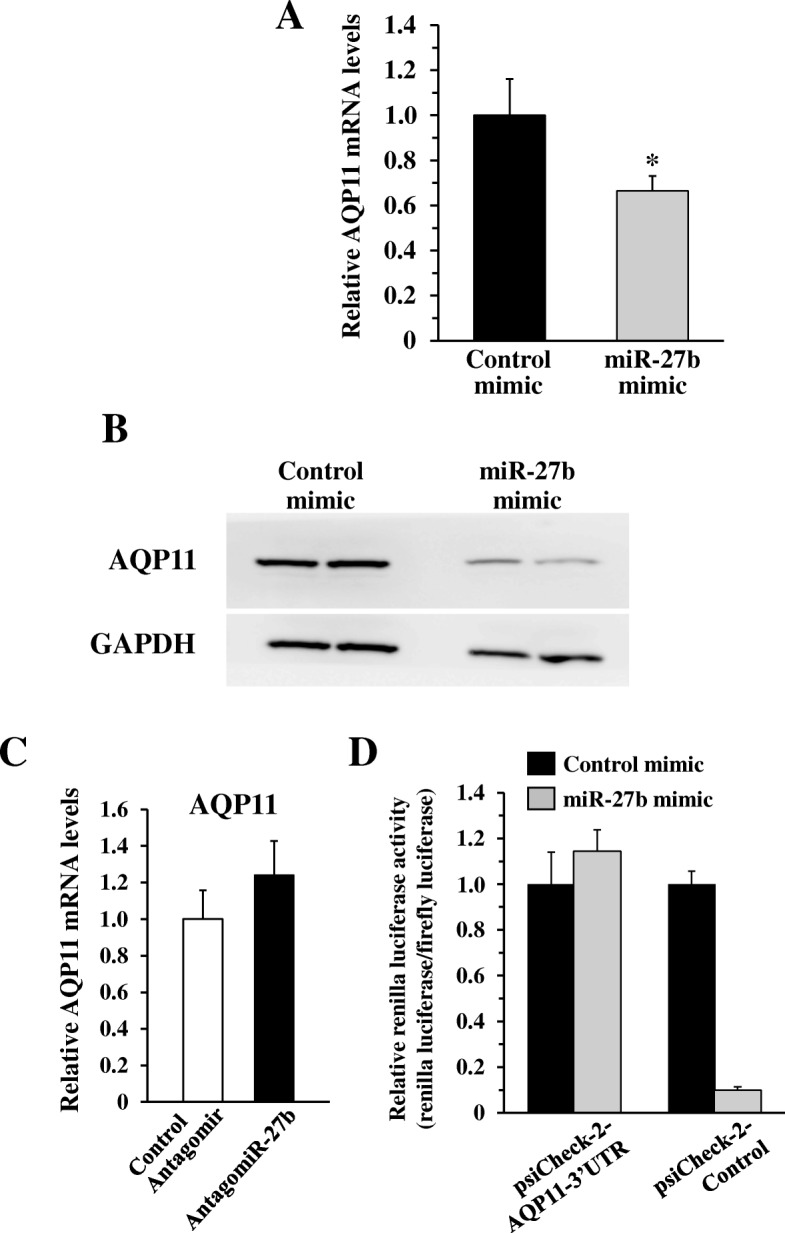
miR-27b-mediated suppression of AQP11 expression in Huh7.5.1 cells. **a** AQP11 mRNA levels in Huh7.5.1 cells following transfection with a miR-27b mimic. Huh7.5.1 cells were transfected with 25 nM of miR-27b mimic or control mimic. Following a 48-h incubation, total RNA was recovered from the cells, followed by real-time RT-PCR analysis. AQP11 mRNA levels were normalized to GAPDH. **b** AQP11 protein levels in Huh7.5.1 cells following transfection with a miR-27b mimic. Huh7.5.1 cells were transfected with 50 nM of miR-27b mimic or control mimic. Following a 72-h incubation, cell lysates were prepared, followed by western blot analysis. Representative image from two independent experiments are shown. **c** AQP11 mRNA levels in Huh7.5.1 cells following transfection with antagomiR-27b. Huh7.5.1 cells were transfected with 10 nM of antagomiR-27b or control antagomir. Following a 48-h incubation, total RNA was recovered, followed by real-time RT-PCR analysis. AQP11 mRNA levels were normalized to GAPDH. D, relative renilla luciferase activities following co-transfection with a reporter plasmid containing the 3′-UTR sequence of the AQP11 gene and miR-27b mimic. HEK293 cells were transfected with 25 nM of miR-27b mimic or control mimic and 1 μg/ml of reporter plasmids. Following a 72-h incubation, renilla and firefly luciferase activities were determined. Renilla luciferase activity levels were normalized to firefly luciferase activity. The data are expressed as the means ± S.D. (N = 3). *p < 0.05

A previous study reported that miR-27b significantly inhibited HCV infection by suppressing the expression of peroxisome proliferator-activated receptor (PPAR)-α and angiopoietin-like protein 3 (ANGPTL3), which are well-known regulators of triglyceride homeostasis [9]. In order to examine whether other target genes of miR-27b regulate HCV infection, we performed in silico screening for novel miR-27b target genes using three miRNA target prediction databases: TargetScan [19], MicroCosm Targets [20], and PicTar [21]. We found 11 miR-27b target gene candidates that were common to all three databases. Among them, we focused on AQP11, because AQP11 was demonstrated to be mainly localized on the ER membrane [22, 23], which is a subcellular organelle important for HCV infection [24]. In addition, there have been no studies reporting the involvement of AQP11 in HCV infection. In order to examine whether the AQP11 gene was a miR-27b target, we transfected Huh7.5.1 cells with a miR-27b mimic. The AQP11 mRNA and protein levels were significantly reduced by the miR-27b mimic (Fig. 2a, b). In contrast, 10 μM of miR-27b antagomir (antagomiR-27b) showed a tendency to increase AQP11 mRNA levels, although not to a statistically significant degree (Fig. 2c). We confirmed that 10 μM of antagomiR-27b significantly up-regulated the mRNA levels of ATP binding cassette transporter A1 (ABCA1), which has been demonstrated to be a miR-27b target gene [25] (data not shown).

Next, we performed a cell-based reporter assay to examine whether miR-27b directly suppressed AQP11 expression via post-transcriptional gene silencing. Co-transfection with a synthetic miR-27b mimic and a reporter plasmid containing two copies of sequences perfectly complementary to miR-27b resulted in approximately 90% reduction in relative renilla luciferase expression levels, compared with co-transfection with a control mimic and a reporter plasmid (Fig. 2d). When a reporter plasmid containing the 3′-UTR of the AQP11 gene was co-transfected, no statistically significant reduction in relative renilla luciferase expression levels was observed. These results suggested that AQP11 was not a direct target gene of miR-27b and that miR-27b indirectly suppressed AQP11 expression.

### Reduction in AQP11 expression resulted in suppression of HCV genome replication

**Fig. 3:**
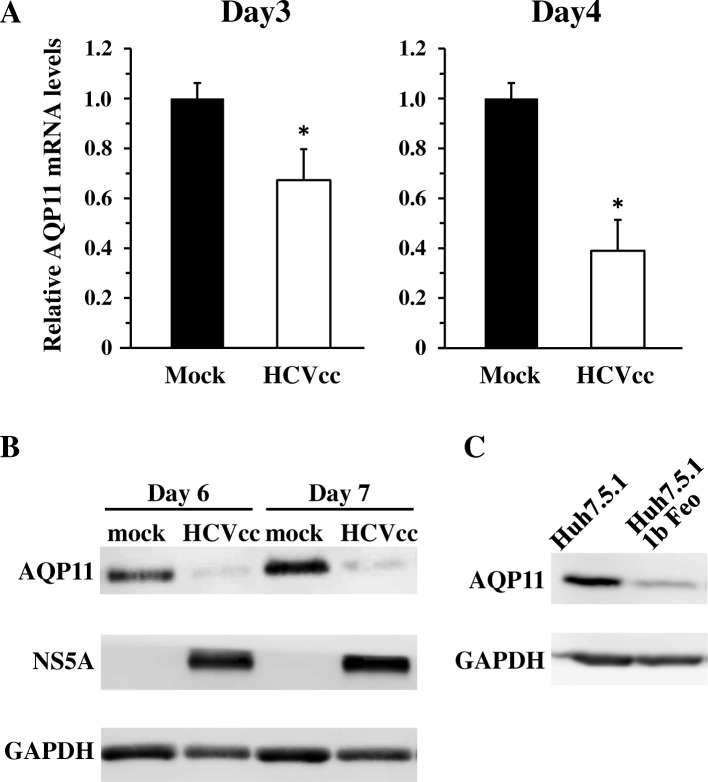
Reduction in AQP11 expression in Huh7.5.1 cells following infection with HCVcc. **a** AQP11 mRNA levels following infection with HCVcc at an MOI of 1. Total RNA was recovered on days 3 and 4, followed by real-time RT-PCR analysis. AQP11 mRNA levels were normalized to GAPDH. The relative AQP11 mRNA levels in mock-infected cells on each day were normalized to 1. The data are expressed as the means ± S.D. (*N* = 3). *p < 0.05. **b** AQP11 protein levels following infection with HCVcc. Huh7.5.1 cells were infected with HCVcc at an MOI of 1. Total cell lysates were prepared at the indicated time points, followed by western blot analysis. **c** AQP11 protein levels in HCV subgenomic replicon-expressing cells. Total cell lysates was prepared from Huh7.5.1 1b Feo cells and Huh7.5.1 cells, followed by western blot analysis. Representative western blot-analysis data from two independent experiments are shown

Next, in order to examine whether HCV infection led to a reduction in AQP11 expression, we determined AQP11 expression levels in Huh7.5.1 cells following infection with HCVcc. AQP11 mRNA levels in HCVcc-infected cells were 31 and 41% lower than those in mock-infected cells on days 3 and 4, respectively, following HCVcc infection (Fig. 3a). There was also an apparent reduction in AQP11 protein levels in Huh7.5.1 cells following HCVcc infection (Fig. 3b). We also examined AQP11 expression levels in HCV replicon-expressing cells, Huh7.5.1 1b Feo cells, to examine whether HCV structural proteins or virus particle production affected AQP11 expression. The HCV subgenomic replicon is a self-replicating HCV RNA sequence, but does not produce progeny virus particles. AQP11 protein levels in Huh7.5.1 1b Feo cells were significantly lower than those in Huh7.5.1 cells (Fig.3c), indicating that HCV structural proteins or virus particle production was not necessary for down-regulation of AQP11 expression. These results indicated that HCV infection resulted in a reduction in AQP11 expression.

**Fig. 4:**
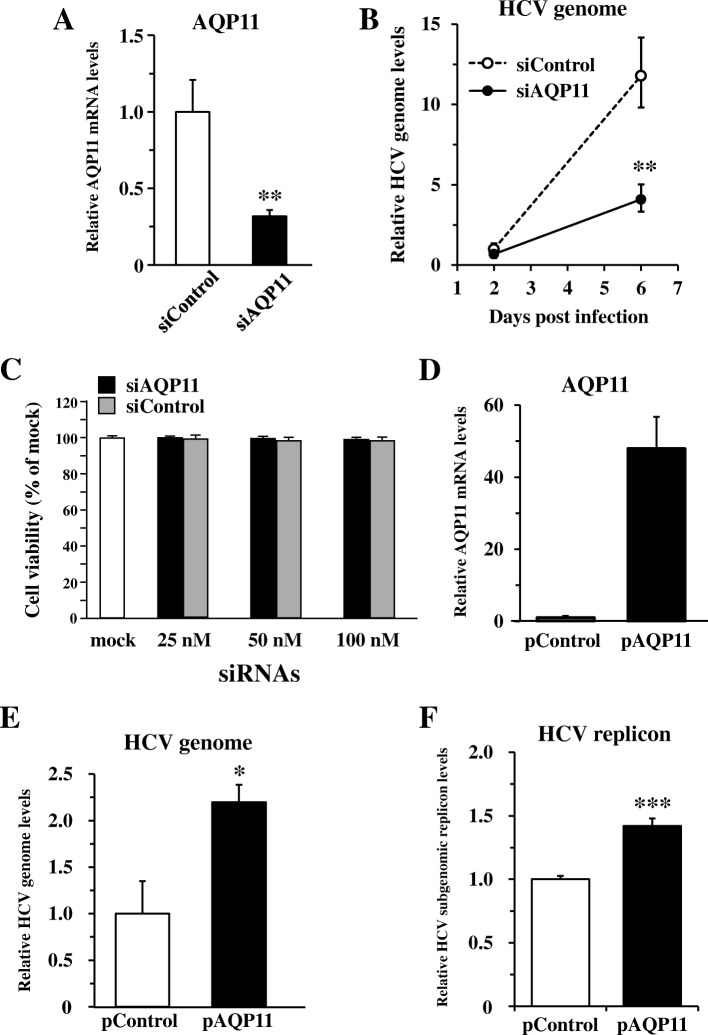
AQP11 is a crucial factor for HCV genome replication. **a** knockdown efficiencies of siAQP11. AQP11 mRNA levels were determined 2 days after siRNA transfection. AQP11 mRNA levels were normalized to GAPDH. **b** HCVcc genome copy numbers in AQP11-knocked down cells. Huh7.5.1 cells were transfected with 50 nM of siAQP11. Two days after siRNA transfection, cells were infected with HCVcc at an MOI of 1. HCVcc genome copy numbers were determined 2 and 6 days after infection. HCVcc genome copy numbers were normalized to GAPDH. The relative HCV genome levels in siControl-treated cells on day 2 were normalized to 1. **c** cell viabilities following knockdown of AQP11. Huh7.5.1 cells were transfected with siAQP11. Cell viabilities were determined 3 days after transfection. The cell viabilities of the mock group were normalized to 100%. **d** AQP11 mRNA levels following transfection with pAQP11. AQP11 mRNA levels were determined 2 days after plasmid transfection. AQP11 mRNA levels were normalized to GAPDH. The relative AQP11 mRNA levels in pControl-treated cells were normalized to 1. **e** HCVcc genome copy numbers in AQP11-overexpressing cells. Huh7.5.1 cells were transfected with 6 μg/ml of pAQP11. Two days after plasmid transfection, cells were infected with HCVcc at an MOI of 0.5. HCVcc genome copy numbers were determined 4 days after infection. The HCVcc genome copy numbers were normalized to GAPDH. Relative HCV genome levels in pControl-treated cells were normalized to 1. **f** HCV subgenomic replicon levels in Huh7.5.1 1b Feo cells following AQP11 over-expression. Huh7.5.1 1b Feo cells were transfected with 6 μg/ml of pAQP11. HCV subgenomic replicon levels were determined 2 days after transfection. The data are expressed as the means ± S.D. (N = 3), *p < 0.05, **p < 0.01, ***p < 0.001

In order to examine whether HCVcc-induced downregulation of AQP11 expression affected HCVcc infection levels, HCVcc was added to AQP11-knocked down Huh7.5.1 cells, followed by real-time RT-PCR analysis of the HCV genome. Transfection with an siRNA against AQP11 (siAQP11) significantly reduced the AQP11 mRNA levels by approximately 70% (Fig. 4a). The HCV genome levels in AQP11-knocked down cells were about 3.2-fold lower than those in control siRNA-transfected cells 6 days after infection (Fig. 4b). Cell viabilities were not significantly altered following AQP11 knockdown (Fig. 4c). HCVcc titers in the culture supernatants or NS5A protein levels in the cells were not apparently altered in AQP11-knockdown cells (data not shown). In contrast, when Huh7.5.1 cells were transfected with a plasmid DNA expressing AQP11 (pAQP11), approximately 47-fold higher levels of AQP11 mRNA were observed, compared with control plasmid-transfected cells (Fig. 4d). The HCV genome levels in AQP11-overexpressing cells were 2.2-fold higher than those in control plasmid-transfected cells (Fig. 4e). These results suggested that suppression of AQP11 expression following HCVcc infection resulted in a reduction in HCV genome copy numbers.

In order to further examine which steps in the HCV infection cycle were inhibited by downregulation of AQP11 expression, AQP11 was over-expressed in Huh7.5.1 1b Feo cells, followed by real-time RT-PCR analysis of the HCV replicon genome. Over-expression of AQP11 resulted in a 1.4-fold increase in HCV subgenomic replicon levels (Fig. 4f). These results suggested that AQP11 was at least partly involved in the HCV genome replication step.

## Discussion

**Fig. 5:**
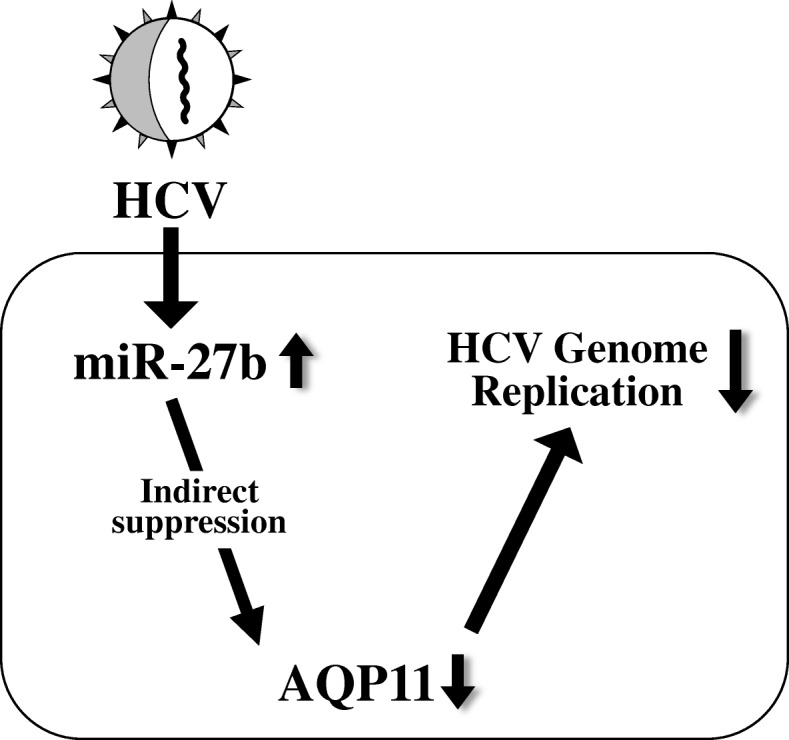
Model of miR-27b-mediated promotion of HCV replication via down-regulation of AQP11 expression. Following HCV infection, miR-27b expression is up-regulated, leading to indirect reduction in AQP11 expression and suppression of HCV genome replication

Previous studies demonstrated that miR-27a/b expression levels were increased following HCV infection and that over-expression of miR-27a/b resulted in suppression of HCV infection [9, 15], suggesting that miR-27a/b functioned as a negative regulator of HCV infection. HCV core and NS4B proteins were involved in induction of miR-27 expression [9]. In the previous studies, miR-27a/b inhibited HCV infection by regulating lipid homeostasis [9, 15, 16], while this study demonstrated that miR-27b indirectly suppressed AQP11 expression, resulting in a reduction in HCV genome replication (Fig.5). The aquaporins (AQPs) are a family of transmembrane channel proteins that are involved in the flow of water, glycerol, and other small molecules across the cellular membranes [26, 27]. The AQP family in mammals is composed of 13 members that are expressed in various tissues. AQP11 is a relatively newly identified AQP family member and is mainly localized on the endoplasmic reticulum (ER) membrane [22, 23], although most of AQP members are expressed on the plasma membrane. The functions of AQP11 in the liver, including the involvement of AQP11 with HCV infection, remain to be clarified, although several studies have reported the functions of AQP11 in the kidney [23, 28, 29]. Madeira et al. demonstrated that AQP11 was preferentially localized in the vicinity of lipid droplets, which are a crucial subcellular organelle for HCV virus particle production, in the adipocytes [30]. Rojek et al. reported that liver-specific AQP11 knockout mice showed vacuolization in the rough ER of periportal hepatocytes after fasting and refeeding challenge [31], suggesting that AQP11 was involved in functions of the ER, which is a subcellular organelle crucial for the HCV life cycle. These findings led us to hypothesize that AQP11 was involved in HCV infection.

Although it remains unclear how AQP11 regulates the HCV life cycle, in our experiments AQP11 over-expression led to an increase in HCV subgenomic replicon copy numbers in Huh7.5.1 1b Feo cells (Fig. 4f), suggesting that AQP11 was involved in the step of HCV genome replication. HCV genome replication occurs in the cytosolic, double-membrane vesicles that are clustered into a membranous web derived from the ER membrane [32, 33]. Reduction in AQP11 expression might lead to a disturbance of ER structure and/or function, thereby suppressing formation of the membranous web and ultimately reducing HCV genome replication. Alternatively, because lipid droplet membranes have been directly linked to viral RNA replication [34], a reduction in AQP11 might result in suppression of HCV genome replication via a disturbance of lipid droplet structure and/or function.

In addition, a reduction in AQP11 expression might inhibit the folding of HCV proteins, leading to suppression of HCV genome replication. mRNA levels of binding immunoglobulin protein (Bip), which is a chaperone protein located in the lumen of ER, were significantly reduced in AQP11-knocked down cells (data not shown). Previous studies demonstrated that Bip protein was interacted with the HCV E1 and E2 proteins [35, 36]. Further analysis will be needed to elucidate the mechanisms of AQP11-mediated regulation of HCV genome replication.

Although AQP11 knockdown resulted in a significant reduction in HCV genome levels in the cells, statistically significant reduction in the virus titers in the culture supernatants was not found following AQP11 knockdown (data not shown). It remains unclear why AQP11 knockdown did not result in significant reduction in the virus titers. AQP11 knockdown indeed mediated a statistically significant reduction in HCV genome levels in the cells, however, the reduction levels were modest (about 3-fold). Virus particle formation or cellular release of virus particles would not be largely enhanced by AQP11 down-regulation.

Several studies have reported a relationship between miR-27b and infection with other types of pathogens, including cytomegalovirus, papillomavirus, and Helicobacter pylori [37–40]. miR-27b augmented the cellular responses against infection with pathogens by suppressing the expression of several target genes, including interleukin (IL)-10, transforming growth factor (TGF)-β-activated protein kinase 1 binding protein 2 (Tab. 2), and Frizzled7 (FZD7) [38, 41]. Several members of the Herpesviridae family express a miR-27 inhibitor for infection [37, 42]. Human adenovirus also suppresses miR-27b expression [40]. miR-27b might function as a host defense system against pathogens. In addition, miR-27b-mediated suppression of the target genes described above might lead to inhibition of HCV infection.

Transfection with a miR-27b mimic resulted in a significant reduction in AQP11 mRNA levels in Huh7.5.1 cells in the present study. On the other hand, a cell-based reporter assay demonstrated that miR-27b did not suppress the expression of a reporter gene containing the 3′-UTR of the AQP11 gene, suggesting that AQP11 was indirectly suppressed by miR-27b. An approximately 270-bp fragment which contained the predicted miR-27b binding sites in the 3′-UTR of the AQP11 gene was inserted into the reporter plasmid. It is possible that miR-27b might bind the other sequences in the 3′-UTR of the AQP11 gene, resulting in a reduction in AQP11 expression, although miR-27b binding sites were not found by TargetScan [19] in the other sequences of the 3′-UTR of the AQP11 gene except for the sequences cloned in the reporter plasmid. Sequences perfectly complementary to miR-27b seed sequences were not found in the coding sequences or 5′-UTR of the AQP11 gene.

In summary, we have demonstrated that miR-27b suppressed AQP11 expression and that HCVcc infection resulted in a significant down-regulation of AQP11 expression, leading to the suppression of HCVcc genome replication, however, reduction in AQP11 expression did not mediate significant effects on HCV titers in the culture supernatants. These findings would provide important clues for elucidation of HCV life cycle and development of antivirus agents.

## Abbreviations

ANGPTL3: Angiopoietin-like protein 3
AQP: Aquaporin
AQP11: Aquaporin 11
Bip: Binding immunoglobulin protein
DAA: Direct-acting antivirus
DMEM: Dulbecco’s Modified Eagle’s medium
ER: Endoplasmic reticulum
FBS: Fetal bovine serum
FZD7: Frizzled7
HCV: Hepatitis C virus
HCVcc: Cell cultured-derived HCV
IFN: Interferon
IL: Interleukin
miRNAs: microRNAs
MOIs: Multiplicities of infection
PPAR: Peroxisome proliferator-activated receptor
TAB2: TGF-β-activated protein kinase 1 binding protein 2
TGF: Transforming growth factor
UTR: Untranslated region
